# Eye care utilization patterns in Tehran population: a population based cross-sectional study

**DOI:** 10.1186/1471-2415-6-4

**Published:** 2006-01-20

**Authors:** Akbar Fotouhi, Hassan Hashemi, Kazem Mohammad

**Affiliations:** 1 Epidemiology and Biostatistics Department, School of Public Health, Tehran University of Medical Sciences, Tehran, Iran; 2 Farabi Eye Hospital, School of Medicine, Tehran University of Medical Sciences, Tehran, Iran; 3 Noor Vision Correction Center, Tehran, Iran

## Abstract

**Background:**

The aim of this study is to determine eye care visits which are an indicator of eye care service utilization by Tehran population and its determinants.

**Methods:**

Through a population-based, cross-sectional study, 6497 Tehran citizens were sampled. All participants had complete eye examinations and an interview regarding demographic and socioeconomic status variables, past medical and eye history, and their previous and last eye care visits.

**Results:**

Among those sampled, 4565 people participated in the study (response rate of 70.3%). Among these participants, 34.7 % had never visited an ophthalmologist or optometrist (95% confidence interval [CI]: 32.4 to 36.9) and 43.2% had not seen an eye care provider in the last 5 years. Multivariate logistic regression revealed that men (OR = 1.30), younger participants (each year increase in age: OR = 0.98) and the less educated (each year increase in education: OR = 0.93) were more likely, and the visually impaired were less likely (OR = 0.41) to have neglected eye care.

**Conclusion:**

A large proportion of the population, including those in the high risk group who require eye care, has never utilized any eye care service. These data suggest that efforts have to be made to better understand the causes and to optimize the utilization of the available eye care services in the population.

## Background

The World Health Organization (WHO) and the International Agency for the Prevention of Blindness have developed a global initiative for the elimination of avoidable blindness by the year 2020; "Vision 2020: the right to sight" [1]. "Vision 2020" includes three major components as target activities: specific disease control, human resource development, and infrastructure and appropriate technology development. The key factors in achieving the goals of "Vision 2020" are eye care services and their utilization. Underutilization of available eye care services and associated factors have been studied in several communities [2-7]. In Iran, ophthalmologists and optometrists are the major eye care providers; general practitioners may provide primary care and refer them to specialists when necessary. Despite the available health care services in Tehran, there is no data concerning their utilization or its associated factors. Providing such information assists in the design of strategies to supply more services to those who have underutilized them. Therefore, in the present study, the history of eye care visits in Tehran population and its associated factors were assessed. Eye care visits provided by ophthalmologists and optometrists were used as a determinant of eye care service utilization

## Methods

The Tehran Eye Study (TES) is a population-based cross-sectional study, and its methodology, described in detail elsewhere [8,9], is presented here briefly. In the TES, a stratified cluster sampling of Tehran Metropolitan Area was used. Selected samples had home interviews and invited to have a complete eye examination and interview at a clinic in Tehran. The eye examinations performed on all participants included visual acuity tests, refraction (objective, subjective and cycloplegic), intraocular pressure measurement, slit lamp examination and fundoscopy. Perimetry was not performed in participants. The information collected at interviews concerned demographic variables, the participants' medical and eye history including previous eye disease, eye trauma, diabetes, hypertension, and also their previous eye care visits. Participants were asked these questions: 'Have you ever seen an eye specialist or optometrist?' and 'When was the last time you visited an eye specialist or optometrist?'.

The study protocol and all the questionnaires have been approved by the corresponding Institutional Review Boards of the Noor Vision Correction Center and the National Medical Research Center of Iran. All participants provided informed consent.

In the analysis, the need for an eye care visit was defined as a presenting visual acuity of worse than 20/40 in the better eye. In calculating the proportions, direct standardization for age and gender was used based on the latest national census in 1996 [10]. In calculating standard errors and 95 percent confidence intervals (95% CI), the cluster sampling design was taken into account and the calculations were adjusted for. Logistic regression analyses were used to explore the factors affecting eye care service utilization. Multivariate logistic regression was fitted based on backward hierarchical elimination approach and the minimal model was reported.

## Results

Of the 6497 enumerated people, a total of 4565 participated in this study and had interviews and examinations from August to December 2002; a response rate of 70.3%. The mean age of the participants was 30.1 years (range 1–96), and 1909 (41.8%) were male. The age and gender distribution of the participants differed from that of Tehran population; those over 40 or under 10 years of age, and women showed a higher participation rate. For this reason, standardizations for age and gender were made based on Tehran population distribution derived from the 1996 national census data. Of those sampled, 2922 (64.0%) participants were 19 years of age or older; among them 35.0%, 4.6%, and 7.5% were employed, unemployed and retired, respectively, 44.5% were housewives, and 8.4%, were students. The mean years of education completed by adult participants was 9.9 years; 9.0% were illiterate and 23.3% had university education.

Of the 4520 participants who responded to questions regarding eye care service use, 34.7% (95%CI: 32.4 – 36.9) had never been examined by an ophthalmologist or optometrist and 40.7% (95%CI: 38.3 – 43.1) had no visit in the last five years (Table 1). These rates both decreased with increasing age. In general, men had a higher rate of having no visit in the last five years, and the univariate analysis showed that the odds ratio (OR) of having no previous eye care visit for men versus women was 1.18 (95%CI 1.03 – 1.34). The multivariate analysis performed after adjusting for age and education showed a greater OR of 1.30 (95%CI: 1.11 – 1.51) for the same comparison (Table 2).

**Table 1:** Distribution of eye care service utilization in Tehran population

	No	No eye care visit Percent (95% CI)	No eye care visit in past 5 years Percent (95% CI)	Odds ratio (95% CI)*
Age				
1 – 19	1716	47.7 (44.9 – 50.9)	46.3 (43.3 – 49.3)^†^	1
20 – 39	1415	29.4 (26.5 – 32.3)	41.3 (38.0 – 44.6)	0.46 (0.40 – 0.52)
40 – 59	1048	23.6 (20.3 – 26.8)	35.0 (31.4 – 38.6)	0.34 (0.30 – 0.41)
60 +	386	12.9 (9.5 – 16.3)	25.3 (20.7 – 29.9)	0.16 (0.12 – 0.22)
Sex				
Women	2656	32.8 (30.0 – 35.6)	40.9 (38.1 – 43.7)	1
Men	1909	36.5 (33.8 – 39.2)	45.3 (42.4 – 48.2)	1.18 (1.03 – 1.34)
Education^‡^				
Illiterate	272	31.0 (25.5 – 36.4)	43.4 (37.0 – 49.8)	1
Primary	784	38.4 (34.6 – 42.3)	44.0 (40.2 – 47.8)	1.39 (1.07 – 1.82)
High school	2444	32.9 (30.4 – 35.5)	43.2 (40.6 – 45.8)	1.09 (0.85 – 1.42)
College	692	16.1 (12.0 – 19.3)	26.0 (22.1 – 29.8)	0.43 (0.30 – 0.61)
Presenting vision in the better eye				
20/20	3200	36.0 (33.5 – 38.5)	44.3 (41.6 – 47.0)	1
20/25–20/40	837	23.7 (20.1 – 27.4)	33.9 (30.0 – 37.8)	0.55 (0.45 – 0.68)
<20/40	350	42.3 (37.5 – 47.2)	49.6 (44.7 – 54.5)	1.31 (1.06 – 1.60)
All	4520	34.7 (32.4 – 36.9)	40.7 (38.3 – 43.1)	

*Univariate logistic regression

‡ Education: illiterate = no formal schooling, primary = 1 – 5 years of schooling, High school = 6 – 12 years of schooling, and college = more than 12 years of schooling

† Calculated for 5 to 19 year old participants

CI = Confidence Interval

**Table 2:** Multivariate odds ratios (95% CI) for never being seen by an eye care provider*

**Independent variables**	**OR (95% CI)**	**P value**
Age (year)	0.98 (0.98 – 0.99)	0.001
Sex (male/female)	1.30 (1.11 – 1.51)	<0.001
Education level (year)	0.93 (0.91 – 0.95)	<0.001
Presenting vision in the better eye		
20/20	1	
20/25–20/40	0.61 (0.49 – 0.76)	<0.001
<20/40	0.41 (0.29 – 0.57)	<0.001

*Multivariate logistic regression

OR = odds ratio, CI = confidence interval

The univariate analysis for the association between a participant's negative history of eye care visits and education is shown in Table 1; more educated people were less likely to have a negative history. Nonetheless, those in the illiterate group had had more visits than those with primary to high school education. Considering the contribution of age to this association and that illiterate people fell into older age groups, multivariate analysis showed a decreasing odds of having no previous eye visit with every one year increase in education (OR = 0.93, CI95%: 91 – 95).

Participants with a visual acuity of worse than 20/40 in the better eye were considered visually impaired and in need of eye care visits. Of the 350 people with visual impairment, 42.3% had no history of an eye examination and 49.6% had no visit in the last five years (Table 1). In a multivariate analysis, the odds of a negative history of eye care visits for these people versus those with normal vision were greater by 1.31. After adjusting for other factors, the odds ratio of a negative history of eye care visits for the visually impaired compared to visually normal people was 0.41 (95%CI 0.29 – 0.57).

Table 2 shows the results of multivariate logistic regression analyses on the association of age, gender, education, and visual impairment with a negative history of eye care visit. Other analyzed factors that were removed from the logistic model were ethnicity, religion, and marital status.

## Discussion

Measuring the effective coverage of health care service is an important part of a health system performance assessment. Assessing health care utilization, which in turn is affected by health care accessibility and individuals' health-seeking behavior, is a conceptual framework for measuring effective coverage of a health care service [11]. In the present study, a history of eye care visit was considered a determinant of eye care service utilization. Results of this study revealed that over one third of the participants had never had an ophthalmic examination, nor had over two fifths of the visually impaired population ever received any eye care service. In a multivariate model we found an increased rate of neglected eye care among younger groups, men, and the less educated, while the visually impaired were more likely to seek eye care. These rates were worse concerning eye care visits in the last five years. We found that, based on their presenting vision, 7.0% of the studied population were visually impaired, among which a considerable 43.2% did not have any eye care visit previously. According to the guidelines issued by the American Academy of Ophthalmology, for those in the 30 to 39 year age group who are free of visual impairment and risk factors, an eye care visit is necessary at least every five years, and the maximum recommended period between eye visits shortens with age; for those over 65 years of age an eye care visit is recommended every 1–2 years [12]. We are far from these guidelines; 43.2% of the visually impaired, and 25.3% of those over 60 years of age have not been examined in the last five years (Table 1), indicating that a considerable proportion of the studied population does not utilize eye care services.

In Iran, Ophthalmology services are available in public hospitals and private sectors at where insurance services cover part of the fees. While Ophthalmologists and optometrists are the major eye care providers, general practitioners may provide primary care and refer them to specialists when necessary. The services are not generally free of charge. Nearly 60% of people are insured by public or private insurance companies. People's income covers a very wide range and the cost of health care especially eye care seems to be high in comparison with the mean income for most people and while eye care services are easily available, it seems some can't afford them.

In different parts of the world, several studies concerning the utilization of eye care services and ophthalmic examinations have been carried out [2-7]. Some studies have focused on particular targets such as the aged [13,14] and diabetics [15,16]. Depending on the geographical variation and the target population, different rates of eye care service utilization have been reported. In a study by Nirmalan and colleagues [2], 64.5% of the target population (rural Indians) never had an eye care visit, while at the other end of the range, Wang et al. [14] have reported a 99% eye care service coverage in an older Australian population. Since these studies are not entirely comparable, it would be difficult to draw logical comparisons.

Some other studies have also found decreasing rates of a negative history with an increase in age [2-4,6,13,14]. Since aging is associated with an increased rate of visual impairment and ophthalmic conditions, a considerable percentage of people are motivated to seek eye care by the factor of need. Results of this study, apart from their etiological importance, exposed a significant proportion of the elderly and visually impaired who have never had an eye care visit or utilized eye care services and must be attended to.

The relationship we found between gender and history of eye care visits coincides with other studies that showed women are more likely to seek eye care [3,4,14]. In some other studies either the reverse was true [2] and men sought eye care more than women, or no significant difference between genders was found [6].

In agreement with other studies, we also found that the greater likelihood of seeking eye care was associated with higher levels of education [2,4,13,14]. This relationship can be attributable to their greater knowledge, and therefore, more reasonable health-seeking behavior. It can also be explained by the fact that educated people are members of the higher socioeconomic class, and may thus have more access to eye care services and find them more affordable.

To mention the limitations of this study, firstly, some factors were not assessed. According to the behavioral model by Anderson [17], factors affecting eye care service utilization can be divided into three categories: predisposing, enabling, and need. In this study, some predisposing factors (demographic variables of age, gender, education, marital status, and religion) and the factor of need were assessed, but not the socioeconomic status or non-behavioral factors such as access to services, their cost, and the attitude of service providers. The extent of the problem in the studied population urges the need for exhaustive studies to identify influential factors more accurately.

Secondly, despite a reasonable response rate of 70.3%, people's access to eye care services may be different between Tehran and the study population. However, it can be assumed that people with eye conditions and those who sought eye care before had a relatively higher participation rate, and so the situation may be even worse in reality.

Thirdly, we were constrained by the sample size and the number of cases for each type of eye disorder to expand our analysis to details and we did not evaluate the utilization of each type of ophthalmic disorder individually. It is worth noting that there is no data about the proportion of people that have been previously diagnosed, but have not utilized care despite the diagnosis.

Fourthly, from a socio-economic point of view, the study sample, consisting of Tehran inhabitants, does not represent the country population, and study results cannot be generalized. It is therefore necessary to carry out similar studies in other parts of the country.

Fifthly, we did not specify whether the eye care provider for each visit was an ophthalmologist or an optometrist. In addition, the study is based on participants' reports which could have been influenced by their judgment on the specialty of the eye care provider or their ability to recall it. Such a limitation does not apply to studies in which data was collected from past records or documenting the providers [6,13].

## Conclusion

Results of this study indicate that a considerable proportion of the studied population had never utilized eye care services; even those at risk and in need of eye care visits. Although not all influential factors were assessed, it is evident that men, the younger age groups, and the less educated are less likely to use these services. These data suggest that efforts have to be made to better understand the causes of eye care service underutilization and to optimize the utilization of the available eye care services in the population.

## Competing interests

The author(s) declare that they have no competing interests.

## Authors' contributions

AF, HH, and KM participated in the design of the study and the preparation of the manuscript. AF and AK participated in the statistical analysis of the study. All authors have read and approved the final manuscript.

## Pre-publication history

The pre-publication history for this paper can be accessed here:


